# A Comparative Evaluation of Frictional Resistance of Various Lingual Brackets With Nitinol (NiTi) Archwires of Different Dimensions: An In Vitro Study

**DOI:** 10.7759/cureus.62121

**Published:** 2024-06-11

**Authors:** Soumya Ranjan Padhi, Shashank Gaikwad, Alok Ranjan, Parag V Gangurde, Harsh Mishra

**Affiliations:** 1 Department of Orthodontics and Dentofacial Orthopedics, Bharati Vidyapeeth (Deemed to be University) Dental College and Hospital, Navi Mumbai, IND; 2 Department of Orthodontics, Bharati Vidyapeeth (Deemed to be University) Dental College and Hospital, Navi Mumbai, IND

**Keywords:** orthodontics, universal testing machine, niti wires, lingual brackets, frictional resistance

## Abstract

Introduction

Orthodontic mechanics involves transferring some of the applied force to the tooth’s supporting components via friction, which in turn allows the tooth to move more easily.

Aim

The purpose of this in vitro experiment was to examine the frictional resistance (FR) of different lingual brackets utilizing Instron universal testing machines and nitinol (NiTi) archwires of varying sizes.

Materials and methods

Twenty-four sectional anterior die stones were replicated from a study model. They were categorized into eight categories, with the Libral lingual bracket and the JJ lingual bracket having 0.012” and 0.014” inch NiTi archwire, which were further subdivided into six subgroups. Three brackets were bonded to the anterior teeth of the upper and lower segments and replicated on other models with the help of silicon putty. Elastomeric modules were ligated to two diameters of NiTi wire (0.012” and 0.014”) in each model. An Instron universal testing machine was used to measure the frictional force. Numerical data and descriptive statistics such as mean and standard deviation have been shown.

Results

In the overall analysis, it was observed that among JJ Orthodontics samples using 0.012” NiTi archwires, the maxilla exhibited a higher FR (4.205N) compared to the mandible (4.092N). Similarly, in the case of Libral Orthodontics samples with 0.012” NiTi archwires, the maxilla also demonstrated a higher FR (5.10N) than the mandible (4.97N). However, this trend did not hold for samples using 0.014” NiTi archwires. There was a statistically nonsignificant difference (p > 0.05) in the values between all the pairs of groups.

Conclusion

The present study concludes that Libral lingual brackets produced overall more FR than JJ lingual brackets.

## Introduction

Lingual orthodontic treatment is one of the methods of treating malocclusion that is designed for patients to have their teeth aligned with an invisible appliance without compromising biomechanical efficiency. There is no doubt that aesthetics is the most important concern for patients, which includes “face,” “teeth alignment,” or “straight teeth” [1]. However, at present, the lingual techniques are practiced by a small group because many orthodontists have found lingual orthodontics difficult to use and limited in application [2]. In lingual orthodontics, the biomechanics differ to a certain extent because of the position of the brackets, i.e., the distance of the bracket to the center of resistance of the tooth, which can lead to a much larger vertical error, especially for the upper incisors. Pierre Fauchard, in 1726, was the first to suggest the use of appliances on the lingual surfaces of the teeth [3]. The concept of lingual orthodontics was first developed in 1967 by Dr. Kinya Fujita, who introduced the use of the lingual multi-bracket system with a mushroom-shaped arch [3]. In 2003, Scuzzo Takemoto bracket was introduced by Giuseppe Scuzzo and Kyoto Takemoto of Japan, which was more comfortable, faster, and more reliable.

William F. Buehler, a research metallurgist at the Naval Ordnance Laboratory in Silver Springs, Maryland (now called the Naval Surface Weapons Center), created the nitinol (NiTi) archwires in the early 1960s with the help of modern appliances [4]. During the first stage of orthodontic treatment, archwires made of this NiTi intermetallic compound level align the teeth thanks to the “memory” phenomenon and the shape memory effect [4]. The frictional force is what the archwire uses when it is ligated to the bracket. It is the force that prevents one item from moving toward another while it is in motion. A material’s surface properties determine the constant value of the coefficient of friction (μ) at the interface. The coefficient of friction multiplied by normal force equals friction. In equation 5, FFR is equal to half of F (N) times µ. In an in vitro experiment, Kusy and Whitley showed that the archwire’s size, form, and size all had a major impact on friction [5,6].

To achieve tooth movement along an archwire, the force applied is essentially the sliding resistance generated in mechanics, which is estimated to be about 50% of the total force applied to the tooth [7]. Hence, the aim of the study is to evaluate and compare the frictional resistance (FR) of various lingual brackets with NiTi archwires of different dimensions in an in vitro environment.

## Materials and methods

Replicating a study model from the Department of Orthodontics and Dentofacial Orthopedics at Bharati Vidyapeeth (Deemed to be University) Dental College and Hospital in Navi Mumbai, India, 24 sectional die stone models were made. Under controlled circumstances, these models were preserved in plastic jars. A complete set of erupted teeth, with crowded anteriors at 21, 22, and 23 and 31, 32, and 33 of normal form and size, was necessary for the research samples. The samples were carefully selected to ensure they were free of carious, hypoplastic, age-related wear teeth, teeth with abnormal anatomy, broken or cracked teeth, and teeth with interproximal restoration. Crowding, as measured by Little’s index, had to be kept between 2 and 3 mm.

**Figure 1:**
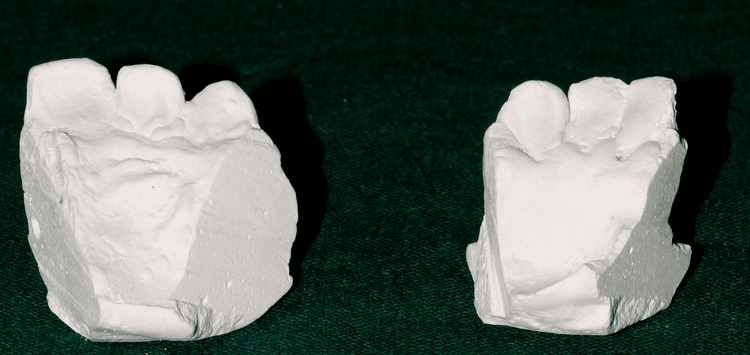
Sectional die stone models of mandibular and maxillary casts

An imprint of the mandibular arch and maxillary arch was taken from a patient who fulfilled all the inclusion criteria when they visited our department. Using this imprint, a mold was created for the research. Each sample of the upper and lower arches’ casts was divided into thirds with the following numbers of teeth: 21, 22, and 23 and 31, 32, and 33 (Figure 1).

**Figure 2:**
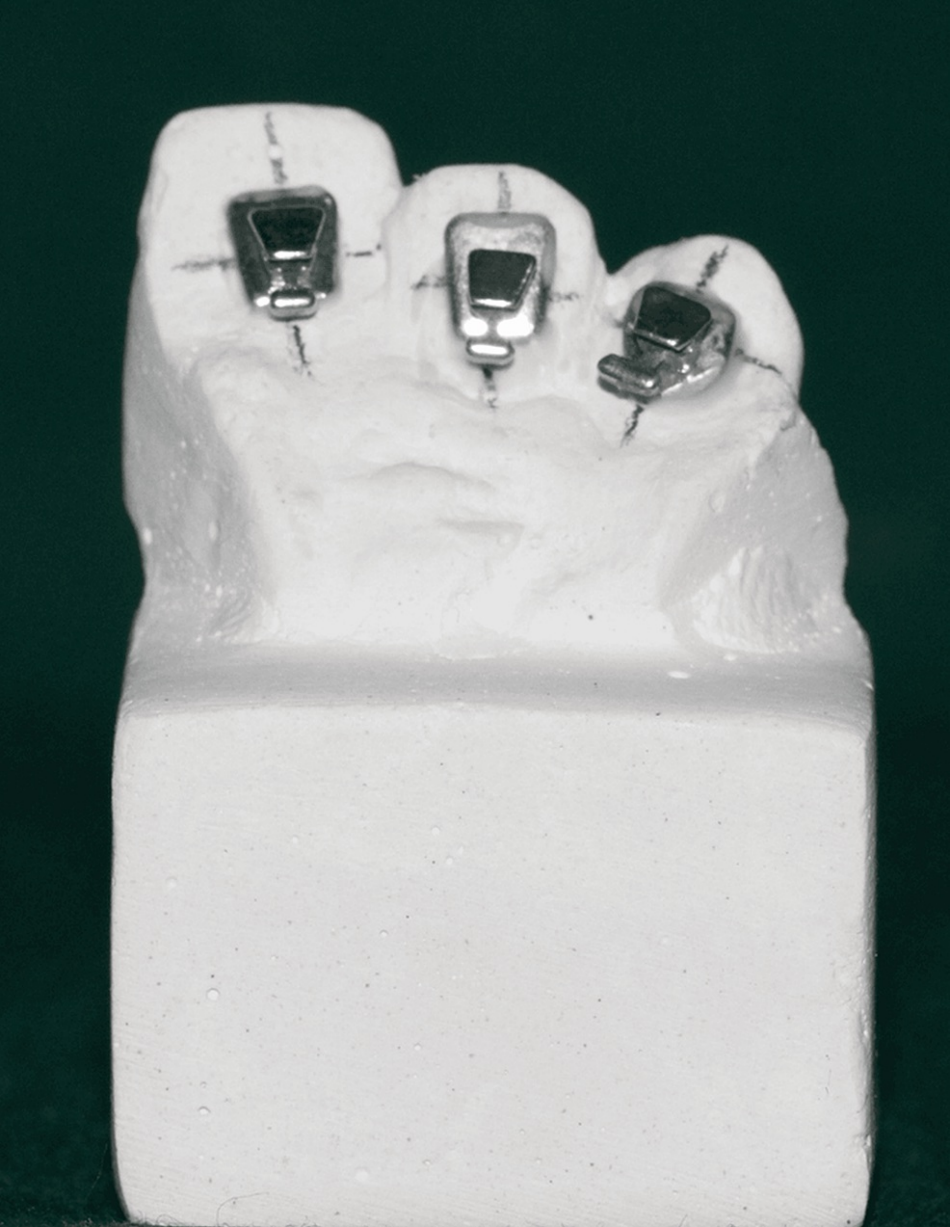
Brackets bonded to the cast

**Figure 3:**
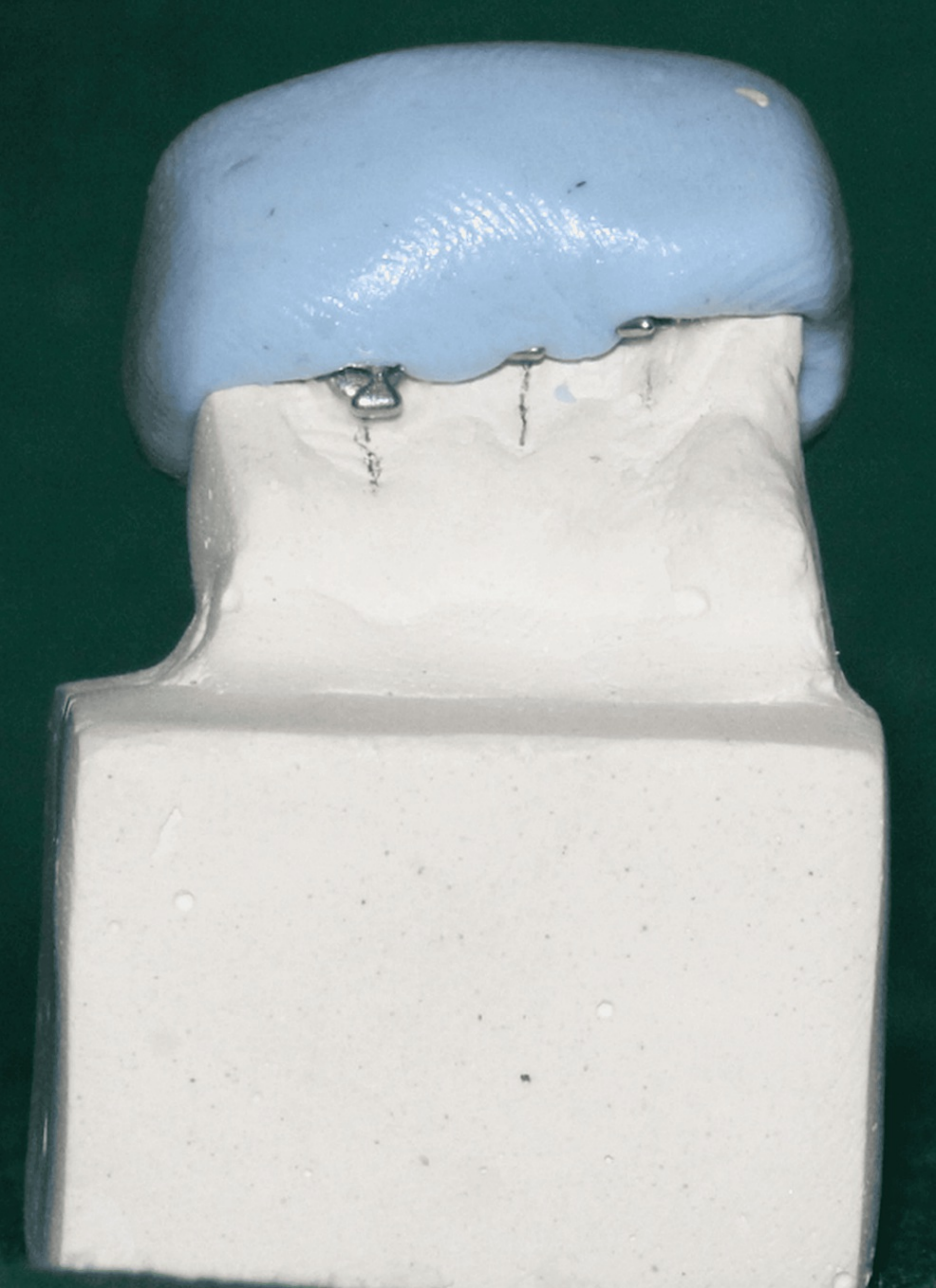
Design and fabrication of a bracket transfer tray utilizing silicone putty

Each of the maxillary and mandibular model bases was constructed using the Leukhart mold. For standardization purposes, silicone putty impressions were taken of both models, which were then repoured into 24 die-stone models with 12 maxillary segments and 12 mandibular segments (Figures 2, 3).

The models were grouped into eight: Group-1(a-f) (JJ mandibular brackets with 0.012” inch NiTi archwire); Group-2(a-f) (JJ mandibular brackets with 0.014” inch NiTi archwire); Group-3(a-f) (JJ maxillary brackets with 0.012” inch NiTi archwire); Group-4(a-f) (JJ maxillary brackets with 0.014” inch NiTi archwire); Group-5(a-f) (Libral mandibular brackets with 0.012” inch NiTi archwire); Group-6(a-f) (Libral mandibular brackets with 0.014” inch NiTi archwire); Group-7(a-f) (Libral maxillary brackets with 0.012” inch NiTi archwire); and Group-8(a-f) (Libral maxillary brackets with 0.014” inch NiTi archwire).

**Figure 4:**
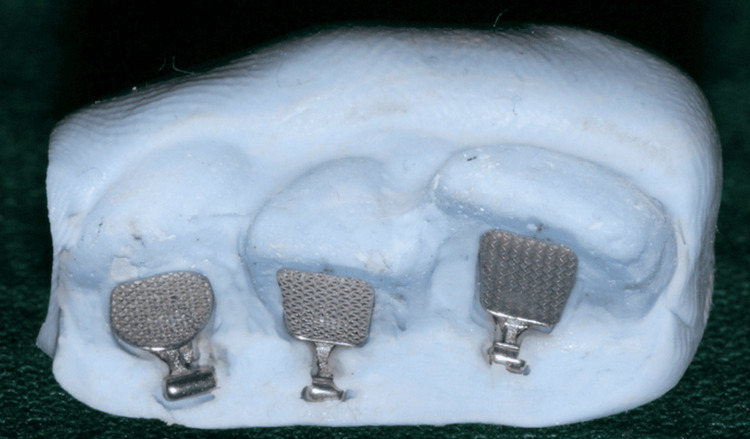
Efficient orthodontic bracket transfer tray

A slot in the clinical crown’s center allowed for the bonding of three brackets to the upper and lower segments’ anterior teeth (Figure 4).

After 30 seconds of etching, the lingual surfaces of the teeth (which were models made of die stone) were cleaned with water for 10 seconds. After 10 seconds, the primer was cured. A layer of composite was placed on the mesh of the brackets, followed by placing the brackets on the models. An excessive flash was removed using a straight probe. The composite was then cured for 20 seconds.

The materials used in this study included Dental Stone Type-IV (Orthokal, Kalabhai), Silicone Putty Regular Set (Aquasil, Dentsply Sirona), Boone’s Gauge (Libral Traders Pvt. Ltd., Mumbai, India), Optic Lingual Bracket (JJ lingual bracket), Libral lingual bracket, and NiTi Wires (JJ). Additionally, Orthoarch Elastomeric Modules, Bracket Placement Tweezer (GDC® Dental Pvt. Ltd., India), Primer (3M Unitek Transbond XT, USA), 37% Ortho-Phosphoric Acid (Discover, USA), and Composite (3M Unitek Transbond XT, USA) were utilized. The curing process was conducted using a Coltolux (Coltene, Switzerland) curing light, and the mechanical testing was performed using an Instron universal testing machine (Instron, Norwood).

**Figure 5:**
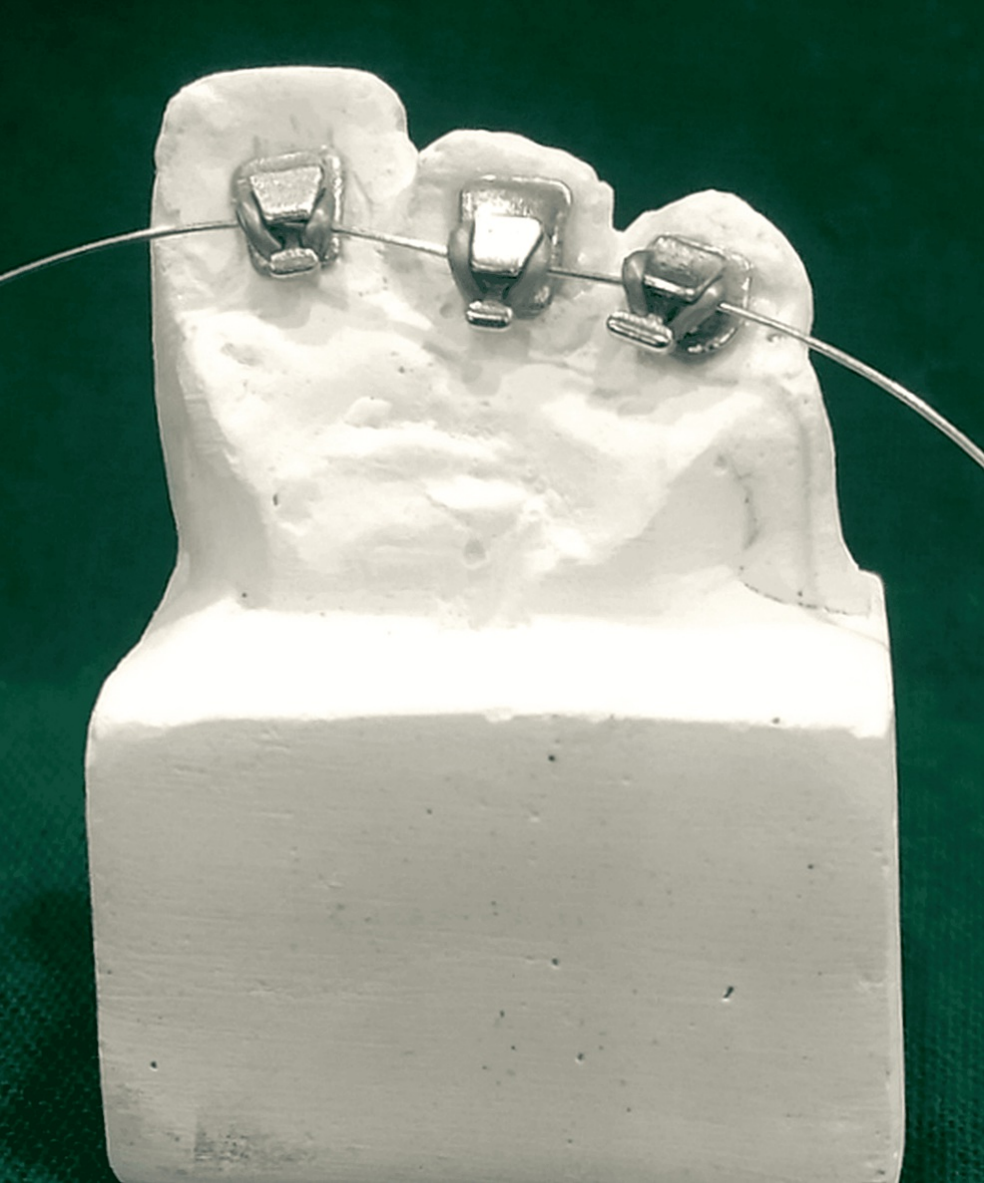
NiTi wire ligature techniques NiTi, nitinol

A silicone putty impression was made with the bracket teeth combination to replicate the exact position of the bracket for further models. Two diameters of NiTi wire, 0.012” and 0.014”, were ligated with elastomeric modules in each model, after which the frictional force was tested (Figure 5).

**Figure 6:**
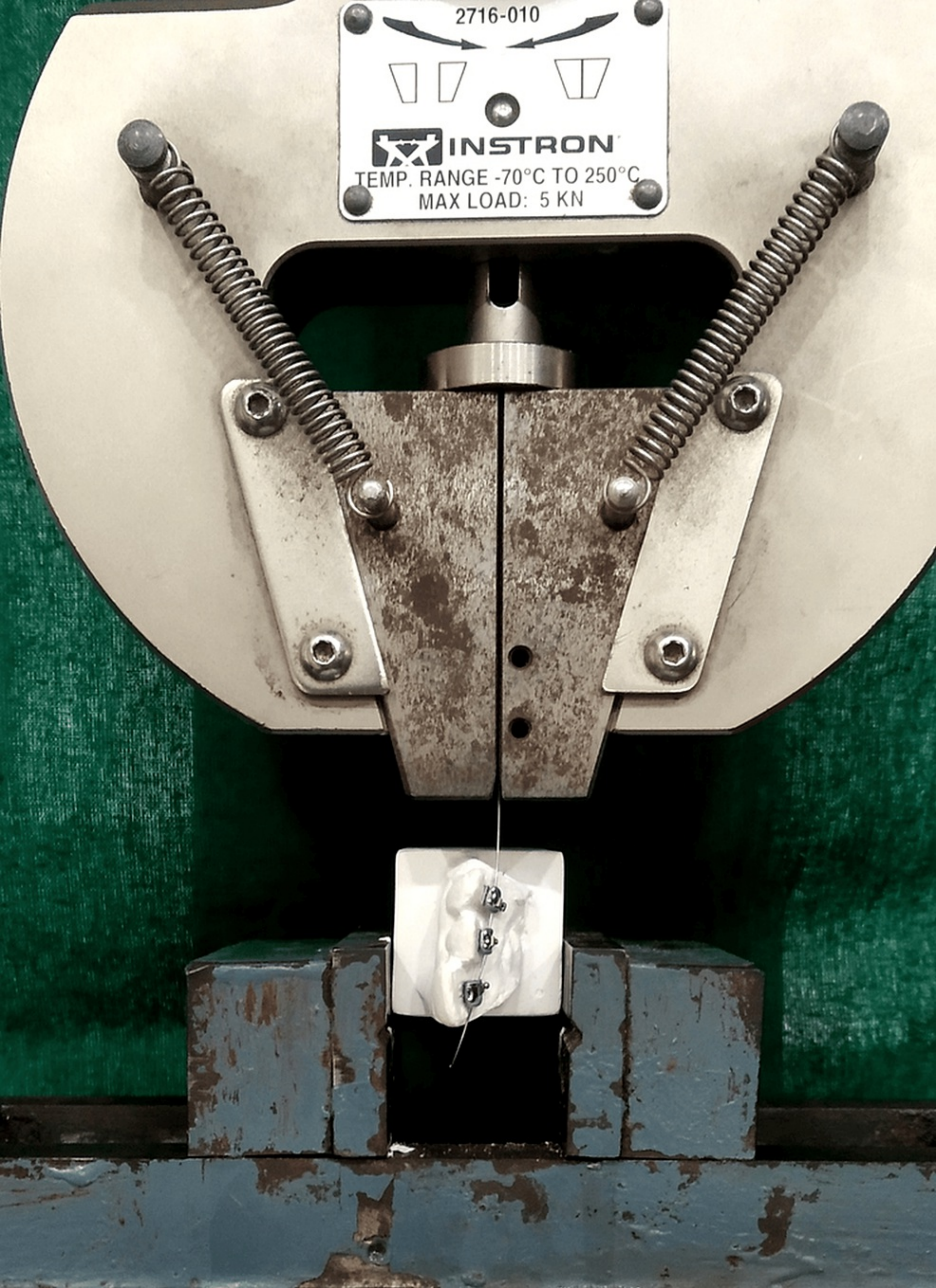
Instron universal testing machine

To find the frictional force, we used an Instron universal testing machine (Maximum Load-5N) to attach the models to the machine in such a way that the wire-pulling force would line with the models’ teeth. A velocity of 1 mm/min for 5 mm was used to ascertain the displacement force of every wire. To recreate the first stage of leveling and alignment, the wire was drawn distantly. Blue Hill Software was used to record the results of the dry-state testing of the bracket/archwire combo on each model (Figure 6).

The statistical methods included coding the data, inputting it into a computer, checking for mistakes, and finally, combining it all into an Excel sheet in Microsoft Office (Microsoft Corporation, Redmond, Washington, United States). IBM SPSS Statistics for Windows, Version 26.0 (Released 2019; IBM Corp., Armonk, NY, USA) was used to do the statistical analysis. Mean, standard deviation, and median were shown as descriptive statistics for numerical data. The sample was able to detect a minimal difference of 0.63 (delta) among the means using a one-way ANOVA test based on a significance threshold of 95% (alpha 0.05). This was accomplished with a power of >80%.

## Results

**Figure 7:**
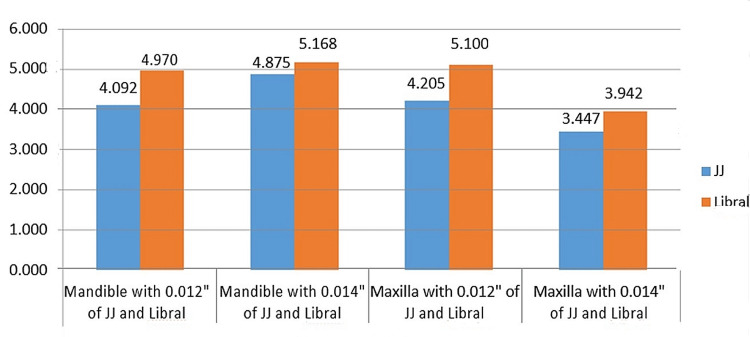
Intergroup comparison of FR between JJ and Libral lingual brackets y-axis: FR (N) FR, frictional resistance

The results of the ANOVA did not reveal any statistically significant variations in the friction measures (p > 0.05). For every kind of bracket, the wire’s size had a major impact on how it slid. In almost all cases, the 0.014 wire generated significantly more friction. All groups’ mean friction values are shown in Figure 7.

In the lower lingual group, the Libral lingual brackets with 0.014” NiTi produced higher friction than other groups. Despite the lack of statistical significance, practical considerations suggest that variations in friction, particularly concerning wire size, could influence treatment mechanics and overall treatment efficiency.

## Discussion

In order to repair the voids left by extractions or move the teeth into proper alignment, orthodontic therapy must overcome FR. Both the present effectiveness and the potential for future repeatability of the orthodontist’s activation force are dictated by the size and variety of the frictional force [8]. Factors such as wire gauge and material, bracket design and material, ligature type and strength, and corporate specifications, which may take the shape of brackets, are considered [9]. In addition, most brackets had flaws in their slots, which would also have contributed to friction [9].

The contact between the archwire and the surface of the bracket creates classical friction. The material-specific properties of archwires and brackets, such as hardness and smoothness, can affect classical friction in three ways. Plowing occurs when a hard material removes part of a softer material, resulting in permanent deformation. Roughing is interlocking due to the engagement of the asperities, which then undergo plastic deformation, after which the movement starts. Binding is the resistance that occurs when the tip of a tooth or wire bends so that the wire contacts the corner of the bracket and binding occurs.

There are three main categories for the numerous approaches to determining the FR between brackets and archwires. One approach is the universal testing machine [10]. The models are secured in one portion of the machine, while another component pulls the wire at a certain contact angle. When the archwire and bracket slot first touch, the angle between them is known as the critical contact angle (θc). The archwire and bracket see a noticeable and quick increase in FR as the angle exceeds this [11]. A fretting machine with an oscillating motion was used in the second way by Willems et al. [12], and a dentoalveolar model, similar to that used with a universal testing machine, was employed in the third technique by Drescher et al. [13] and Loftus et al. [14]. The frictional force between orthodontic archwires and brackets was assessed in research conducted by Nishio et al. [15]. For two minutes at a pace of half a centimeter per minute, we examined the magnitude of the frictional forces produced by ceramic brackets, ceramic brackets with a metal-reinforced slot, and stainless steel brackets combined with various archwires.

In the present study, a larger value of FR was found with an increase in the diameter of the wire, except in Group 4 and Group 8. In a previous study by Almeida et al. [16], anterior segments of the maxilla and mandible were taken. Based on the result, they concluded that the larger the wire diameter, the larger the value of friction, which was also observed in this study. Even a study by Ozturk et al. [9] concluded the same observation. In the present study, examining the results of various archwire/bracket combinations, it was found that the FR in the JJ lingual bracket on the mandible with 0.012” and the JJ lingual bracket on the mandible with 0.014” was 4.09N and 4.87N, respectively. When the evaluation was done between the groups comprised of the Libral lingual bracket on the mandible with 0.012”, it was found that the FR was 4.97N, whereas with the group of Libral lingual bracket on the mandible with 0.014”, the FR was 5.16N. The group comprises the JJ lingual bracket on the maxilla with 0.012”; it was found that the FR is 4.2N, whereas the group of the Libral lingual bracket on the maxilla with 0.012” had an FR of 5.1N. When the evaluation was done on the JJ lingual bracket on the maxilla with 0.014", the FR was 3.44N, whereas with the Libral lingual bracket on the maxilla with 0.014”, it was 3.9N. In the present study, examining the results of various archwire/bracket combinations, it was interesting to note that the FR of Group 6 (Libral lingual bracket on the mandible with 0.014”) was the highest among all groups (SD = 1.731), whereas the stress at the maximum load was highest for Group 7 (Libral lingual bracket on the maxilla with 0.012”).

Libral Orthodontics FR outperformed JJ Orthodontics in research comparing the two brands of lingual brackets, which used data from both the maxilla and the mandible. Additionally, the way the wire was kept in the slot had a major impact on slipping. Consistent with prior research linking the archwire dimension to friction, the current study found that, relative to the mandible, frictional forces were proportional to archwire sizes. However, with respect to the maxilla and mandible samples of JJ Orthodontics with 0.012” NiTi archwires, the maxilla had a higher FR (4.205N) than the mandible (4.092N), and with respect to Libral Orthodontics with 0.012” NiTi archwires, the maxilla also had a higher FR (5.10N) than the mandible (4.97N), but this did not hold true with 0.014” NiTi archwires. On further comparison, we could see that in Groups 4 and 8, there was reduced FR (3.4N and 3.94N, respectively) with both brands of lingual brackets when pulled with 0.014” NiTi archwire. This observation was similar to the study by Matarese et al. [5], where they did the study on conventional labial brackets and self-ligating brackets, where a significant reduction of friction was found in the latter group. The reason for this might be that it enhances the efficiency of using the wires' mechanical qualities during the alignment step.

In contrast to earlier research, we examined the friction that forms during the first phases of treatment using round (nickel titanium 0.014 and 0.012) wires rather than rectangular ones. During the alignment and leveling process, it is best to employ small-diameter archwires that are spherical to enhance elasticity and reduce slide mechanics-related friction [14]. FR values were not significantly different across groups in this study (p > 0.05) for all variables; however, this finding may have clinical significance and aid the clinician in selecting the best bracket/archwire combination for a particular ligation technique.

The findings of this study underscore the importance of considering the diameter and material of archwires, as well as the type and brand of brackets used, in orthodontic treatments. The observed differences in FR between various combinations of archwires and brackets highlight the need for orthodontists to carefully select the most appropriate materials to optimize treatment efficiency. Specifically, the study demonstrates that larger wire diameters generally result in higher FR, and the choice between JJ and Libral lingual brackets can significantly impact the FR, especially during the initial alignment phases. These insights can guide clinicians in making informed decisions to reduce friction, enhance the effectiveness of orthodontic forces, and improve patient outcomes. The choice of archwire diameter and bracket type significantly affects FR, with larger diameters generally increasing FR. Both JJ and Libral brackets showed variable performance depending on the wire size and location. These findings emphasize the importance of tailored bracket and wire selection to optimize orthodontic treatment efficiency and effectiveness.

The study has several limitations to consider. Firstly, the use of plaster models lacks the physiological function of teeth in vivo, where support comes from the periodontal ligament and alveolar bone. Secondly, since friction is influenced by numerous factors, determining the precise cause of changes in friction is challenging. Additionally, while all bracket/archwire combinations were meticulously tested, the evaluation was limited to only two quadrants (one upper and one lower). When analyzing the data, it is important to keep in mind that these constraints could impact how applicable the conclusions are to a broader context.

## Conclusions

The study found that larger arch wires result in a higher FR. This FR, present during leveling, space closure, and torque control stages of treatment, can have both beneficial and detrimental effects. Additionally, Libral lingual brackets exhibited a higher total FR compared to JJ lingual brackets. However, the differences in FR between the various group pairings were not statistically significant (p > 0.05). Further research is needed to evaluate how different archwire factors affect frictional force in lingual brackets.
